# Mannose phosphate isomerase gene mutation leads to a congenital disorder of glycosylation: A rare case report and literature review

**DOI:** 10.3389/fped.2023.1150367

**Published:** 2023-04-12

**Authors:** Siliang Lu, Shuheng Liang, Yi Wu, Jinyi Liu, Lin Lin, Guosheng Huang, Huaijun Ning

**Affiliations:** Department of Pediatrics, Guangxi Clinical Research Center for Pediatric Diseases, Maternal and Child Health Hospital of Guangxi Zhuang Autonomous Region, Nanning, China

**Keywords:** mannose phosphate isomerase, congenital disorder of glycosylation, MPI-CDG, carbohydrate deficient glycoprotein syndrome, CDG

## Abstract

We report the case of a 2-year-old girl who was diagnosed with Mannose-6-phosphate isomerase-congenital disorder of glycosylation (MPI-CDG) and provide a review of the relevant literature. The young girl presented with recurrent unexplained diarrhea, vomiting, hypoproteinemia, and elevated liver transaminases. Whole-exome sequencing revealed that the patient had compound heterozygous mutations in the MPI gene (NM_0024). An exon 4 (c.455G > T, p.R152l) mutation was inherited from the mother and an exon 7 (c.884G > A, p.R295H) mutation from the father. One week after the start of mannose treatment, the vomiting and diarrhea symptoms disappeared completely and did not show any side effects. We also provide a brief review of the relevant literature. Including the present case, a total of 52 patients from hospitals across 17 countries were diagnosed with MPI-CDG. Age at disease onset ranged from birth to 15 years, with an onset under 2 years in most patients (43/50). Overall, patients presented with at least one or more of the following symptoms: chronic diarrhea (41/46), vomiting (23/27), hepatomegaly (39/44), hepatic fibrosis (20/37), protein-losing enteropathy (30/36), elevated serum transaminases (24/34), hyperinsulinemic-hypoglycemia (24/34), hypoalbuminemia (33/38), prolonged coagulation (26/30), splenomegaly (13/21), non-pitting edema (14/20), failure to thrive (13/36), portal hypertension (4/9), epilepsy (2/17), thrombosis (12/14), and abnormally elevated leukocytes (5). None of the patients was reported to have an intellectual disability (0/28). The majority of patients (26/30) showed clinical symptoms, and laboratory results improved after oral mannose administration. Our findings suggest that MPI-CDG should be considered in children with unexplained recurrent digestive and endocrine systems involvement, and gene examination should be performed immediately to obtain a definite diagnosis in order to begin treatment in a timely manner.

## 1. Introduction

Congenital disorders of glycosylation (CDG) are rare genetic disorders caused by defects in glycoprotein and glycolipid glycan synthesis and attachment. CDG were first reported by the Belgian scholar Jaak Jaeken in 1980 (1). According to the different step localizations of glycosylation defects, CDG were originally classified into two types (I and II) (2). Given that glycosylation is involved in a variety of biological processes, the clinical phenotype in patients diagnosed with CDG is highly heterogeneous, ranging from asymptomatic to mild symptoms to severe multiple organ involvement (3).

Mannose-6-phosphate isomerase-congenital disorder of glycosylation (MPI-CDG) is caused by mutations in MPI genes. As of August 23, 2022, fewer than 60 cases had been reported, and no more than three cases had been reported in China. The main symptoms in patients with MPI-CDG are chronic diarrhea, cyclic vomiting, hypoglycemia, coagulation abnormalities, protein-losing gastrointestinal disease, and congenital hepatic fibrosis (4), which do not involve the nervous system. Currently, there are several approaches to the diagnosis of MPI-CDG: gene analysis, isoelectric focusing of transferrin, high-performance liquid chromatography, and capillary electrophoresis. However, due to our poor understanding of the disease, as well as limitations in technology, MPI-CDG is often misdiagnosed as pediatric enteritis or a milk protein allergy in infancy and early childhood. When left undiagnosed and untreated, the patient's prognosis is often poor. Notably, there is currently only one effective treatment available for MPI-CDG *via* oral mannose supplementation (5).

In this study, we reviewed the clinical manifestations, genetic characteristics, and laboratory examinations of a child with MPI-CDG admitted to our institution and reviewed the relevant literature to increase awareness of the disease among clinicians.

## 2. Case report

A 2-year-old girl was admitted to the emergency department with diarrhea for 4 days and vomiting and fever for 3 days. Four days before admission, the patient developed diarrhea, manifested as a yellow watery stool with small amounts of blood and mucus 5–8 times per day, followed by vomiting and fever. Vomiting occurred once to several times daily, and one important inducement was diet, with no bile, blood, or coffee-ground material present. The patient presented with intermittent fever, and the highest body temperature recorded was 39.5°C. The patient had no other accompanying symptoms. Since the onset of symptoms, she reported a worse mental condition, unsatisfactory appetite, decreased urination, and slightly reduced body weight.

She had been hospitalized for diarrhea on four occasions since the age of 4 months. She was hospitalized at 4 months (other hospitals), 11 months (other hospitals), 1 year and 11 months (our hospital), and 2 years (our hospitals). After treatment with intravenous fluids, the patient was discharged after her condition improved. The patient was the first child of the family and was spontaneously delivered after a full-term pregnancy with a birth weight of 2,600 g. The patient and her father were diagnosed with β-thalassemia, and her mother was healthy. There was no other significant associated history, and there was no history of similar symptoms among other family members.

Upon physical examination, she was in poor general condition and appeared emaciated. Her height and weight were below the 3rd percentile, but her mental and motor developmental steps were normal. Her skin was dry, thin, and had scanty subcutaneous fat, with signs of dehydration. Her spleen and liver were not palpable below the costal margins. Slightly visible pitting edema was found in both lower extremities, and other physical examinations revealed no abnormalities.

After admission, relevant laboratory and imaging examinations were completed; the specific results are shown in Table 1. The remaining renal function and etiology tests, including respiratory virus tests; stool, blood, and cerebrospinal fluid cultures; and other laboratory examinations, showed no abnormalities. The imaging findings of the patient are described below. The results from a Doppler ultrasound revealed the following: (1) an enhanced echo of the liver parenchyma and uneven echoes; (2) the gallbladder wall was thick and edematous; and (3) fluid in the abdominal perisplenic area. Notably, no abnormalities were observed in gastric or intestinal tissues.

**Table 1:** The main laboratory findings during two hospitalizations in our hospital.

Age at onset and hospital days	1 year and 11 months (Prior admission, 7 days)		2 years and 1 month (Current admission, 10 days)		Normal range
	Admission	Discharge	Admission	Discharge	
WBC	22.2	18.7	22.2	12.9	5.1–14.1 × 109/L
N	36.7	41	69.5	35.1	13%–55%
HGB	104	103	103	97	>110 g/L
MCV	60.4	61.9	60.5	64.4	72–86 fl
MCHC	299	302	308	285	310–355 g/L
hs-CRP	11.19	1.88	15.08	0.78	0–10 mg/L
ALT	13	18	49	34	<30 µ/L
AST	47	57	171	88	<44 µ/L
TP	38.07	42.96	31.75	50.95	61–76 g/L
ALB	25.61	29.29	20.44	36.26	39–54 g/L
IgG	3.493	/	2.772	/	5.0–13.0 g/L
IgA	0.298	/	0.413	/	0.4–1.8 g/L
IgM	0.492	/	0.434	/	0.4–1.8 g/L
APTT	40	/	48.7	/	23.0–36.0 s
INR	1.05	/	1.08	/	0.8–1.20
Insulin	/	/	19.6	/	17.8–173 pmol/L
Glucose	4.8	5.4	5.8	4.9	3.9–6.1 mmol/L
Factor II	/	/	/	138.6	70%–120%
Factor VIII	/	/	/	69.1	70%–150%
Factor IX	/	/	/	59.6	70%–120%
Protein C	/	/	/	94.6	70%–140%
Protein S	/	/	/	30.6	59%–118%
Gene sequencing	/	/	c.455G > T c. 884G > A	/	-

The symbol “/” indicates this ancillary analysis was not performed. WBC, white blood cells; N, neutrophils; HGB, hemoglobin; MCV, mean corpuscular volume; MCHC, mean corpuscular hemoglobin concentration; hs-CRP, high-sensitivity C-reactive protein; ALT, alanine transaminase; AST, aspartate transaminase; TP, total protein; ALB, albumin; APTT, activated partial thromboplastin time; INR, international normalized ratio.

Colonoscopy was also performed. Biopsies obtained from the ileocecal region and sigmoidal curve revealed mild chronic inflammation. Before the results of the genetic analysis were provided, her condition was diagnosed as pediatric enteritis based on the clinical course and examination results.

We performed whole exome sequencing on the patient and Sanger sequencing on DNA from the parents. Sequencing analysis revealed two heterozygous missense mutations in MPI, c.455G > T (p.R152l) and c. 884G > A (p.R295H). Sanger sequencing confirmed this result and revealed that the two heterozygous mutations were inherited from the patient's parents. According to the ACMG guidelines, c.455G > T (p.R152l) was classified as a likely pathogenic variant. No similar variants have previously been reported in the literature, and there are no results of pathogenicity in the ClinVar database. Grading Standards: PM2_Supporting + PM3 (Trans)+PM5(4) +PP3_Strong. The allele frequency of the variants is 0.0000106 at gnomAD. Conversely, c. 884G > A (p.R295H) was classified as a pathogenic variant. Grading Standards: PM2_Supporting (0.0000412) +PM3_Strong (13) +PP3_Strong. The allele frequency of the variants is 0.0000318 at gnomAD. Based on these findings, the patient was diagnosed with a congenital disorder of glycosylation MPI-CDG.

Therefore, the following therapeutic measures were first administered: fluid therapy (oral rehydration solution and intravenous fluids), anti-infective therapy (z/sulbactam sodium), conventional liver protection drugs, probiotic therapy (glutathione), amino acid formula feeding, supplemental human albumin, and trace elements (zinc). After 1 week of treatment, the diarrhea and vomiting were significantly relieved, and she no longer exhibited a fever. The results of the laboratory tests are shown in Table 1. At this point, the patient's family requested discharge. Therefore, the patient did not receive mannose therapy during hospitalization. After discharge, the parents were telephoned for follow for up to 4 weeks; she was started on oral mannose on the day following hospital discharge. The dose was 150 mg/kg/dose four to five times a day, administered orally. One week after the start of mannose treatment, the vomiting and diarrhea symptoms disappeared completely and did not show any side effects. The parents of the patient reported high satisfaction with the mannose therapy and the gains made during treatment (**Statement**: The benefits and risks of the treatment were explained to family members prior to the administration of Mannose treatment, who indicated their understanding of the situation.).

## 3. Literature review

We performed a systematic review of the literature on August 23, 2022, using the PubMed, Embase, Web of Science, CNKI, Wanfang, and Weipu databases. The following keywords were used: “CDG-Ib;” “MPI”; “MPI-CDG”; “congenital disorder of glycosylation type Ib”; “mannose phosphate isomerase”; “congenital disorder of glycosylation Ib”; AND “congenital disorder of glycosylation” OR “carbohydrate deficient glycoprotein syndrome”. No language or data filter was used in this study, nor were any search filters for the study type, language, or date of publication. Our search yielded a total of 94 articles. In total, 31 articles were excluded from our analysis after removing duplicate articles, review articles, and articles that did not meet our standards. Each article was reviewed, and the results were summarized. However, not every article provided basic information about MPI-CDG patients, clinical symptoms, or routine laboratory tests.

Excluding the present case, a total of 51 cases of MPI-CDG have been reported in the literature. Eleven cases came from France, six each from the Netherlands and Turkey, four from America, three each from Canada and Poland, two each from Egypt, Germany, Russia, Spain, China, Denmark, and Asia, and one each from Australia, Sweden, Caucasus, and the United Arab Emirates. After preliminary analysis, there were 15 male patients and 28 female patients. The age at disease onset ranged from birth to 15 years, with an onset under 2 years in most patients (43/50).

**Figure 1:**
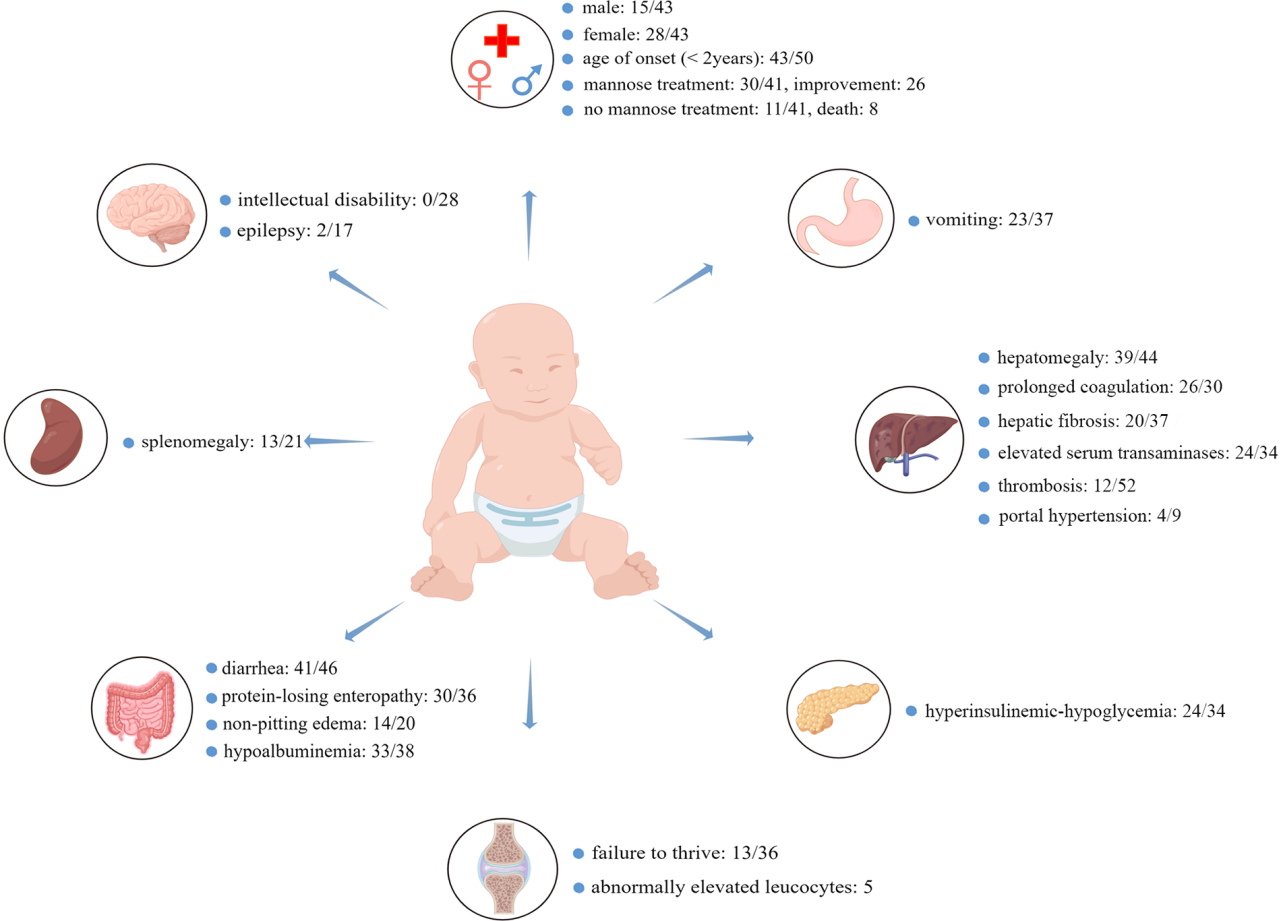
The most common clinical symptoms and abnormal laboratory findings in MPI-CDG. In total, 52 cases were identified, and the age of onset of MPI-CDG was usually under 2 years of age. MPI-CDG typically presents with single or multi-organ involvement, commonly involving digestive and endocrine systems, but not the nervous system. Since not every article provided basic information about the MPI-CDG patients, clinical symptoms, or routine laboratory tests, the number of cases varied between studies, and uninformative cases were not counted in the total. For example, diarrhea: 41/46 means that 41 patients had diarrhea, five patients did not, and relevant information was not provided for six patients.

As shown in Figure 1, almost all patients had gastrointestinal or hepatic involvement, with the main presenting signs being chronic diarrhea (41/46), vomiting (23/27), hepatomegaly (39/44), hepatic fibrosis (20/37), and protein-losing enteropathy (30/36), but none of them developed intellectual disability (0/28). Notably, these patients had varying degrees of abnormalities in laboratory results, such as elevated serum transaminases (24/34), hyperinsulinemic-hypoglycemia (24/34), hypoalbuminemia (33/38), or prolonged coagulation (26/30). Some patients had splenomegaly (13/21), non-pitting edema (14/20), failure to thrive (13/36), portal hypertension (4/9), epilepsy (2/17), thrombosis (12/14), and abnormally elevated leukocytes (5). Importantly, not all patients with MPI-CDG had abnormal clinical manifestations or laboratory examinations (see Supplementary Table S1 for details). Of the 41 patients, 30 received mannose therapy, and 26 showed significant improvement. Of the 11 patients who did not receive mannose therapy, eight died.

In our analysis, the three most frequently reported mutation sites were c.656G > A, c.884G > A, and c.1193T > C, followed by c.455G > A, c.655C > T, c.636G > A, c.419T > C, and c.413T > C. The base-change type c.455G > T observed in our case report has not yet been reported in the literature. The detailed results are listed in Supplementary Table S2, and the detailed clinical characteristics and laboratory findings for all patients are described in Supplementary Table S1.

## 4. Discussion

In the present case, the patient was admitted to the hospital because of diarrhea. Her age of onset was 4 months, and diarrhea was also the first clinical symptom. Notably, the age of the patient was similar to that reported in the literature review, as disease onset occurred below 2 years of age in the majority of the patients (43/50). MPI-CDG usually involves the digestive and endocrine systems, and it presents with a “classic triad” consisting of hepatic (liver fibrosis, hepatopathy), gastroenterological (diarrhea, protein-losing enteropathy), and endocrine (hyperinsulinemic-hypoglycemia) involvement (6), but the clinical picture varies among cases. This phenomenon might be because MPI enzyme activity varies among MPI-CDG patients. In fact, these patients typically have hypomorphic mutations in MPI, resulting in decreased, but not total, loss of MPI activity (7). Diet is another possible explanation, as some plants and fruits, such as cranberries, contain abundant mannose, and when patients include these foods in their diet, it is possible to reduce their symptoms.

**Figure 2:**
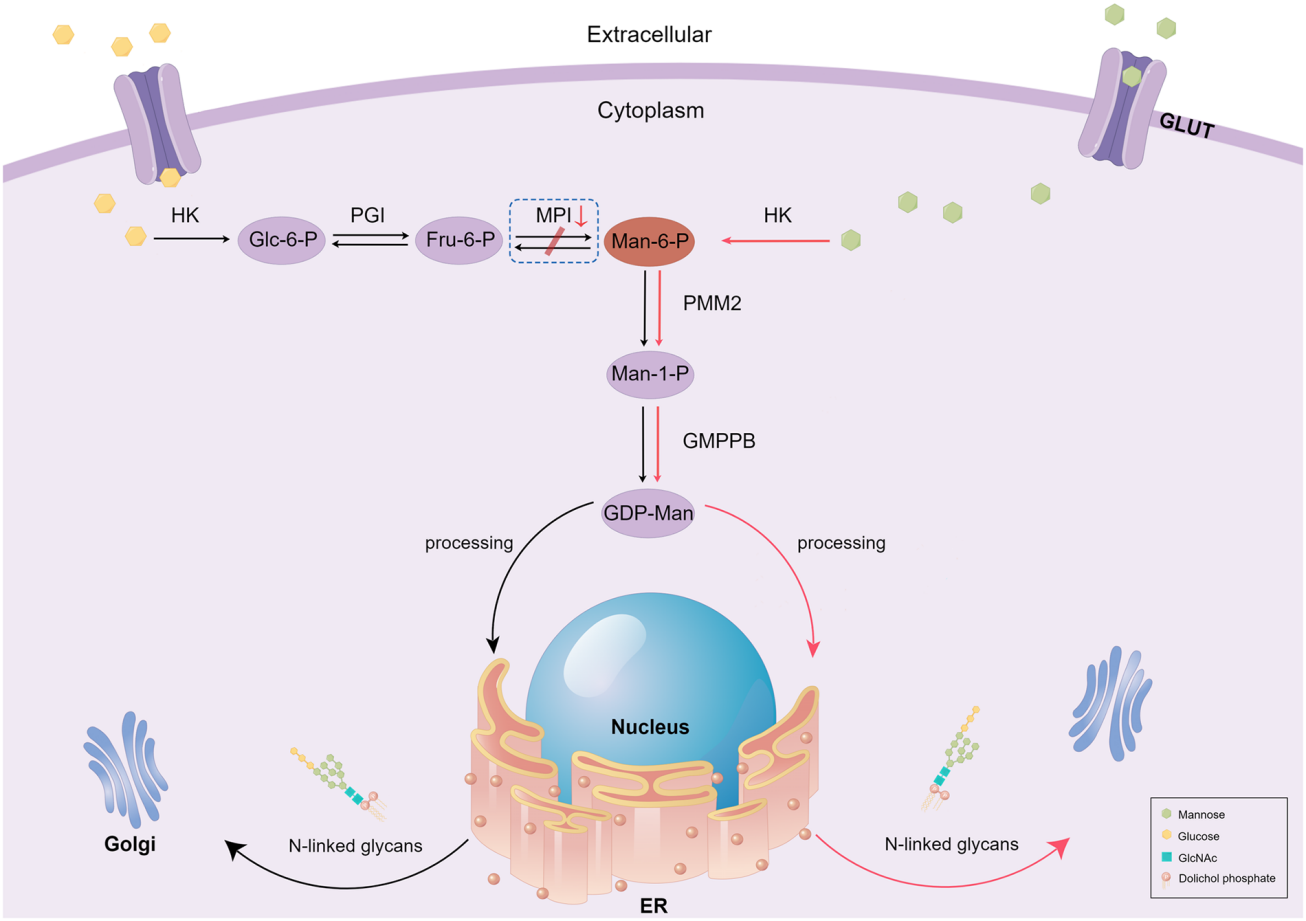
Schematic diagram of mannose metabolism and therapy in MPI-CDG patients. In this figure, we only present the part related to the MPI defects. Black arrows represent normal mannose metabolism, and the box with dashed lines represents when the process goes wrong. Red arrows represent the mechanism underlying exogenous mannose's therapeutic action in MPI-CDG. GULT, glucose transport; HK, hexokinase; Glc-6-P, glucose-6-phosphate; PGI, phosphoglucose isomerase; Fru-6-P, fructose-6-phosphate; MPI, mannose phosphate isomerase; Man-6-P, mannose-6-phosphate; PMM2, phosphomannomutase 2; Man-1-P, mannose-1-phosphate; GMPPB, GDP-mannose pyrophosphorylase B; ER, endoplasmic reticulum; Golgi, Golgi apparatus; GlcNAc, N-acetyl glucosamine.

Based on our literature review, hepatomegaly (39/44) and diarrhea (41/46) are the most common symptoms of MPI-CDG. Protein glycosylation mainly occurs in the cytoplasm, endoplasmic reticulum, and Golgi apparatus, and eight different biochemical pathways are involved in this process (2). Depending on where the glycosylation defect occurs, CDG is divided into three categories: defects in protein N-glycosylation, defects in protein O-glycosylation, and defects in multiple glycosylation pathways. MPI-CDG is classified as a protein N-glycosylation defect, as the lack of MPI precludes the interconversion of mannose 6-phosphate (Man-6-P) and fructose 6-phosphate (Fru-6-P), which eventually affects the N-glycosylation process (Figure 2). The most common type of glycosylation that occurs in mammalian proteins is N-glycan linkage (8), which contributes to the correct folding and stability of nascent proteins. In addition, glycan can affect protein sorting and delivery in the cell interior. In MPI-CDG patients, abnormal N-glycan linkage causes a conformational change in the newly synthesized proteins, which creates unstable proteins that lead to functional changes. It is well known that the liver is the main source of protein synthesis in organisms, including the synthesis of albumin, antithrombin III, fibrinogen, and lipoprotein. Hepatic steatosis has been reported in the MPI-CDG liver tissue (9), and abnormal lipoprotein transport induced by CDG might be the cause of this phenomenon. This may also explain why patients with MPI-CDG usually show liver impairments. The corresponding clinical manifestations and laboratory findings include hepatomegaly, hepatic fibrosis, elevated serum transaminase levels, hypoproteinemia, and prolonged coagulation. In our patient, the symptoms were similar to those mentioned above, with the exception of hepatomegaly and hepatic fibrosis (Table 1). However, we suspected that this patient had liver fibrosis because liver echo enhancement and inhomogeneity were the common ultrasonographic findings of liver fibrosis (we did not perform a hepatic biopsy because the patient's parents did not give consent). Beyond this, our patient did not show other related symptoms, such as splenomegaly and portal hypertension, which may be due to the various stages and severity of the disease.

Another common symptom in patients with MPI-CDG is diarrhea, including in the present case, with most patients experiencing diarrhea as the initial symptom (9–13). In these patients, there is a close link between diarrhea and protein-losing enteropathy. Normally, the intestinal barrier consists of enterocytes and intercellular tight junctions, preventing the loss of proteins from the gastrointestinal tract. However, abnormal glycosylation can affect the function of the intestinal barrier. In fact, when the barrier is disrupted or transmucosal permeability is increased (14), leakage of plasma proteins, such as albumin, into the gastrointestinal tract can cause secretory diarrhea, which is another possible cause of hypoproteinemia. According to the colonoscopy examination and pathology, intestinal inflammation was also present in our patient, which may have aggravated the loss of albumin from the gastrointestinal tract. In the current case, the patient presented with ascites, gallbladder wall edema, and slightly visible pitting edema in both lower extremities caused by hypoproteinemia. For this reason, we need to pay attention to MPI-CDG, which is easily misdiagnosed as celiac disease.

In addition to hepatic and gastrointestinal disorders, MPI-CDG is often accompanied by endocrine system impairments, affecting the levels of multiple hormones (15). The most common presentations were hyperinsulinemic-hypoglycemia (24/34). Insulin secretion is controlled by several factors, including nutrients, paracrine processes, and the autonomic nervous system (16). However, all these factors are linked to K^+^ ATP channels, and some substances such as glucosum, sulfonylureas, and amino acids (leucine) can inhibit this channel, thus enabling the release of insulin (17). Previous studies have shown that the sulfonylurea receptor (SUR1) in K^+^ ATP channels is a glycoprotein (18); therefore, hyperinsulinism in CDG may be a consequence of defective glycosylation of this protein. However, some patients with MPI-CDG showed a favorable response to diazoxide (19). Nevertheless, more interaction studies are required to confirm this possibility. Another study found that patients with CDG exhibited marked hyperplastic islets accompanied by an increase in β-cell numbers (20), which triggers high insulin levels, resulting in severe hypoglycemia. Taken together, this suggests that glycosylation disorders can adversely affect the endocrine system.

In addition to these common presenting symptoms, a number of other clinical signs are worth highlighting. These include venous thrombosis and abnormally elevated leukocyte levels. In fact, among the 52 patients with MPI-CDG, 12 had a history of thrombosis, including jugular vein thrombosis, deep venous thrombosis, and intracranial thrombosis (6, 21, 22). In humans, the liver produces the majority of coagulation factors. Importantly, aberrant glycosylation can lead not only to hypocoagulability but also to hypercoagulability. Therefore, attention should be paid to this point when treating patients with MPI-CDG to prevent venous thrombotic events. Another common symptom of MPI-CDG is abnormally elevated leucocytes. We noticed a strongly elevated white blood cell count of 22.2 × 10^9^/L in the current case, with a predominance of neutrophils. This finding is in agreement with the results of Vuillaumier-Barrot et al. (13). We speculate that the reason for this phenomenon is that the cell adhesion molecules associated with glycosylation were affected. Neutrophils exist in the blood circulatory system (circulating pool), vascular wall (marginal pool), and bone marrow (storage pool). White cell counts reflect the number of neutrophils in the circulating pool. In a normal physiological scenario, the circulating pool and marginal pool exist in a dynamic equilibrium, and upon inflammation, leukocytes from the storage pool enter the peripheral blood and migrate to the injured site. In fact, interactions between leukocytes and the vascular endothelium are crucial for leukocyte rolling, adhesion, and extravasation, and this process is mediated by cell adhesion molecules, such as L-selectin, E-selectin, and vascular cell adhesion molecules. When MPI-CDG occurs, the expression of adhesion molecules may also affect the interaction between vascular cells and leukocytes, causing an increase in the number of circulating leukocytes. Therefore, it is necessary to determine if there is an inflammatory response. Finally, we also found that the patient had mild hypochromic microcytic anemia; there could be two possible explanations for this. First, she has a history of β-thalassemia and second, nutritional iron deficiency anemia caused by gastrointestinal inflammation and long-term diarrhea induced by MPI-CDG.

The diagnosis and treatment of MPI-CDG have been previously presented in detail by Čechová et al. (23). Briefly, MPI-CDG can be successfully treated by oral mannose supplementation, as exogenous mannose can bypass the deficiency caused by MPI that contributes to N-glycan synthesis through Man-6-P (Figure 2). International consensus guidelines recommend oral administration of mannose at a concentration of 150–170 mg/kg bodyweight, four to five times per day (24). A review of the literature revealed that of 30 patients who received mannose therapy, 26 showed improved clinical symptoms and laboratory results. The biochemical and clinical symptoms usually disappear in less than 2 weeks in addition to liver injury. Conversely, out of the 11 patients that did not receive mannose treatment, eight died during the follow-up period. Therefore, early detection, diagnosis, and mannose therapy treatment are especially important for improving the prognosis and quality of life of patients with MPI-CDG.

The patient's characteristics in the present case were as follows: ① recurrent attacks of diarrhea and vomiting reported since infancy; ② elevated liver enzymes and prolonged coagulation; ③ hypoproteinemia and edema with proteins likely lost through the gastrointestinal tract; ④ a heterozygous missense mutation in exons 4 (c.455G > T) and 7 (c.884G > A) of the MPI gene that came from the parents separately, consistent with autosomal recessive inheritance; and ⑤ abnormally elevated leukocytes with no significant endocrine system involvement. The underlying mechanism(s) of the condition are unknown; thus, whether it is linked to the locus of gene mutation or other factors requires further research. Based on the clinical manifestations and genetic testing results, the patient was diagnosed with MPI-CDG.

## 5. Conclusion

MPI-CDG is a rare autosomal recessive inherited disease, with onset in early childhood in most patients, which is caused by an MPI gene mutation that leads to a dramatic decrease in MPI enzymatic activity, thus affecting N-glycan linkage. MPI-CDG may present with single- or multi-organ involvement, commonly involving the digestive and endocrine systems but not the nervous system. The main clinical symptoms are vomiting, diarrhea, hepatomegaly, elevated serum transaminases, and hypoalbuminemia, with or without endocrine system abnormalities, such as hyperinsulinemic-hypoglycemia. Currently, the diagnosis of MPI-CDG relies on genetic testing, and it can be effectively treated with oral exogenous mannose. Based on our findings, we recommend that MPI-CDG should be considered in children with the above symptoms, and a genetic examination should be performed immediately to obtain a definite diagnosis and ensure the timely initiation of effective treatment.

## Author contributions

SL and SL collected clinical data, performed follow-up investigations, and drafted the manuscript. YW, JL, and LL analyzed the results of whole exome sequencing, enteroscopy, ultrasonography, and pathology; contributed to the discussion; and reviewed and edited the manuscript. SL, SL, and GH performed the literature review, performed data collection and management, analyzed data, and reviewed and revised the manuscript. HN followed the patient; designed, conceptualized, and executed data analyses and interpretations; and reviewed and revised the manuscript. All authors contributed to the article and approved the submitted version.

## References

[1] Jaeken J. Congenital disorders of glycosylation. Ann N Y Acad Sci. (2010) 1214:190–8. 10.1111/j.1749-6632.2010.05840.x 21175687

[2] Hennet T Cabalzar J. Congenital disorders of glycosylation: a concise chart of glycocalyx dysfunction. Trends Biochem Sci. (2015) 40(7):377–84. 10.1016/j.tibs.2015.03.002 25840516

[3] Pérez-Cerdá C Girós ML Serrano M Ecay MJ Gort L Pérez Dueñas B A population-based study on congenital disorders of protein N- and combined with O-glycosylation experience in clinical and genetic diagnosis. J Pediatr. (2017) 183:170–7.e1. 10.1016/j.jpeds.2016.12.060 28139241

[4] Schollen E Dorland L de Koning TJ Van Diggelen OP Huijmans JG Marquardt T Genomic organization of the human phosphomannose isomerase (MPI) gene and mutation analysis in patients with congenital disorders of glycosylation type ib (CDG-ib). Hum Mutat. (2000) 16(3):247–52. 10.1002/1098-1004(200009)16:3<247::AID-HUMU7>3.0.CO;2-A 10980531

[5] Jones MA Ng BG Bhide S Chin E Rhodenizer D He P DDOST mutations identified by whole-exome sequencing are implicated in congenital disorders of glycosylation. Am J Hum Genet. (2012) 90(2):363–8. 10.1016/j.ajhg.2011.12.024 22305527 PMC3276676

[6] Mühlhausen C Henneke L Schlotawa L Behme D Grüneberg M Gärtner J Mannose phosphate isomerase deficiency-congenital disorder of glycosylation (MPI-CDG) with cerebral venous sinus thrombosis as first and only presenting symptom: a rare but treatable cause of thrombophilia. JIMD Rep. (2020) 55(1):38–43. 10.1002/jmd2.12149 32905087 PMC7463055

[7] Chu J Mir A Gao N Rosa S Monson C Sharma V A zebrafish model of congenital disorders of glycosylation with phosphomannose isomerase deficiency reveals an early opportunity for corrective mannose supplementation. Dis Model Mech. (2013) 6(1):95–105. 10.1242/dmm.010116 22899857 PMC3529342

[8] Reed UC. Congenital muscular dystrophy. Part II: a review of pathogenesis and therapeutic perspectives. Arq Neuropsiquiatr. (2009) 67(2a):343–62. 10.1590/S0004-282X2009000200035 19547838

[9] Abdel Ghaffar TY Ng BG Elsayed SM El Naghi S Helmy S Mohammed N MPI-CDG from a hepatic perspective: report of two Egyptian cases and review of literature. JIMD Rep. (2020) 56(1):20–6. 10.1002/jmd2.12159 33204592 PMC7653262

[10] Haznedar P Eminoğlu FT. An overlooked case of a treatable hyperinsulinemic hypoglycemia: congenital glycosylation defect type ib. Turk Pediatri Ars. (2020) 55(1):79–81. 10.5152/TurkPediatriArs.2018.18004 32231455 PMC7096565

[11] Kelly DF Boneh A Pitsch S Gold H Fietz M Nelson P Carbohydrate-deficient glycoprotein syndrome 1b: a new answer to an old diagnostic dilemma. J Paediatr Child Health. (2001) 37(5):510–2. 10.1046/j.1440-1754.2001.00671.x 11885720

[12] Noman K Hendriksz CJ Radcliffe G Roncaroli F Moreea S Hussain A Clinical outcomes in an adult patient with mannose phosphate isomerase-congenital disorder of glycosylation who discontinued mannose therapy. Mol Genet Metab Rep. (2020) 25:100646. 10.1016/j.ymgmr.2020.100646 32963965 PMC7490551

[13] Vuillaumier-Barrot S Le Bizec C de Lonlay P Barnier A Mitchell G Pelletier V Protein losing enteropathy-hepatic fibrosis syndrome in saguenay-lac st-jean, Quebec is a congenital disorder of glycosylation type ib. J Med Genet. (2002) 39(11):849–51. 10.1136/jmg.39.11.849 12414827 PMC1735008

[14] Levitt DG Levitt MD. Protein losing enteropathy: comprehensive review of the mechanistic association with clinical and subclinical disease states. Clin Exp Gastroenterol. (2017) 10:147–68. 10.2147/CEG.S136803 28761367 PMC5522668

[15] Zdemir TR. Congenital disorder of glycosylation: clinical and molecular characteristics of 9 patients from Turkey. J Behcet Uz Child Hosp. (2020) 10(3):267–73. 10.5222/buchd.2020.09471

[16] Gosmain Y Cheyssac C Masson MH Guérardel A Poisson C Philippe J. Pax6 is a key component of regulated glucagon secretion. Endocrinology. (2012) 153(9):4204–15. 10.1210/en.2012-1425 22778220

[17] Panten U Burgfeld J Goerke F Rennicke M Schwanstecher M Wallasch A Control of insulin secretion by sulfonylureas, meglitinide and diazoxide in relation to their binding to the sulfonylurea receptor in pancreatic islets. Biochem Pharmacol. (1989) 38(8):1217–29. 10.1016/0006-2952(89)90327-4 2650685

[18] Bernardi H Fosset M Lazdunski M. Characterization, purification, and affinity labeling of the brain [3H]glibenclamide-binding protein, a putative neuronal ATP-regulated K+ channel. Proc Natl Acad Sci U S A. (1988) 85(24):9816–20. 10.1073/pnas.85.24.9816 3144003 PMC282872

[19] Babovic-Vuksanovic D Patterson MC Schwenk WF O'Brien JF Vockley J Freeze HH Severe hypoglycemia as a presenting symptom of carbohydrate-deficient glycoprotein syndrome. J Pediatr. (1999) 135(6):775–81. 10.1016/S0022-3476(99)70103-4 10586187

[20] Sun L Eklund EA Chung WK Wang C Cohen J Freeze HH. Congenital disorder of glycosylation id presenting with hyperinsulinemic hypoglycemia and islet cell hyperplasia. J Clin Endocrinol Metab. (2005) 90(7):4371–5. 10.1210/jc.2005-0250 15840742

[21] Girard M Douillard C Debray D Lacaille F Schiff M Vuillaumier-Barrot S Long term outcome of MPI-CDG patients on D-mannose therapy. J Inherit Metab Dis. (2020) 43(6):1360–9. 10.1002/jimd.12289 33098580

[22] Tamminga RY Lefeber DJ Kamps WA van Spronsen FJ. Recurrent thrombo-embolism in a child with a congenital disorder of glycosylation (CDG) type ib and treatment with mannose. Pediatr Hematol Oncol. (2008) 25(8):762–8. 10.1080/08880010802394616 19065443

[23] Čechová A Altassan R Borgel D Bruneel A Correia J Girard M Consensus guideline for the diagnosis and management of mannose phosphate isomerase-congenital disorder of glycosylation. J Inherit Metab Dis. (2020) 43(4):671–93. 10.1002/jimd.12241 32266963 PMC7574589

[24] Park JH Marquardt T. Treatment options in congenital disorders of glycosylation. Front Genet. (2021) 10(12):735348. 10.3389/fgene.2021.735348 PMC8461064 34567084

