# Supplementary material for: Mannose phosphate isomerase gene mutation leads to a congenital disorder of glycosylation: A rare case report and literature review
Source: Front Pediatr. 2023 Apr 12;11:1150367. doi: 10.3389/fped.2023.1150367 (PMC10130505; doi:10.3389/fped.2023.1150367)
Supplement: Supplementary file 1 [file Datasheet1.docx]

Supplementary Material

# Supplementary Tables

**Supplementary Table 1.** Clinical characteristics and laboratory findings of patients with MPI-CDG

| Patient number | | 1^[1]^ | 2^[1]^ | 3^[2]^ | 4^[2]^ | 5^[2]^ | 6^[2]^ | 7^[3]^ |
| --- | --- | --- | --- | --- | --- | --- | --- | --- |
| General information | Years of publication | 1980 | 1980 | 1985 | 1985 | 1985 | 1985 | 1998 |
|  | Country | DK | DK | FR | CA | CA | CA | GER |
|  | Gender | M | F | NM | NM | NM | NM | M |
|  | Age of onset | 2.5 y | 2.5 y | 2 w | 2 w | 2m | Born | 11m |
| Presenting symptom | Diarrhea | Yes | Yes | Yes | Yes | Yes | Yes | Yes |
|  | Vomiting | NM | Yes | Yes | Yes | Yes | Yes | Yes |
|  | Nonimmune  Hydrops | Yes | Yes | Yes | Yes | Yes | Yes | Yes |
|  | Hepatomegaly | Yes | Yes | Yes | Yes | Yes | Yes | Yes |
|  | Splenomegaly | NM | NM | NM | NM | NM | NM | NM |
|  | Portal hypertension | No | No | NM | NM | NM | NM | NM |
|  | Failure to thrive | NM | NM | NM | NM | NM | NM | No |
|  | Intellectual disability | NM | NM | NM | NM | NM | NM | No |
|  | Seizures | NM | NM | No | No | No | Yes | NM |
|  | Protein-loosing enteropathy | Yes | Yes | Yes | Yes | Yes | Yes | Yes |
| Laboratory findings | Hepatic fibrosis | Yes | Yes | Yes | Yes | Yes | Yes | NM |
|  | Elevated serum transaminases | NM | NM | NM | NM | NM | NM | Yes |
|  | Hypoalbuminemia | Yes | Yes | Yes | Yes | Yes | Yes | Yes |
|  | Hyperinsulinism | NM | NM | NM | NM | NM | NM | NM |
|  | Hypoglycemia | NM | NM | Yes | Yes | Yes | Yes | Yes |
|  | Prolonged coagulation | Yes | Yes | Yes | Yes | Yes | Yes | Yes |
|  | Base change 1 | NM | NM | c.884G>A | c.884G>A | c.884G>A | c.884G>A | c.656G>A |
|  | Protein change 1 | NM | NM | R295H | R295H | R295H | R295H | R219Q |
|  | Base change 2 | NM | NM | c.884G>A | c.884G>A | c.884G>A | c.884G>A | c.656 G>A |
|  | Protein change 2 | NM | NM | R295H | R295H | R295H | R295H | R219Q |
|  | Other | Thrombosis | No | High WBC | High WBC | High WBC | High WBC | Thrombosis |
| Mannose treatment |  | No | No | No | No | No | No | Yes |
| Outcome |  | Death | Death | NM | Death | Death | Death | Better |

**Supplementary Table 1 (continued)**

| Patient number | | 8^[4]^ | 9^[4]^ | 10^[4]^ | 11^[5]^ | 12^[6]^ | 13^[7]^ | 14^[7]^ |
| --- | --- | --- | --- | --- | --- | --- | --- | --- |
| General information | Years of publication | 1998 | 1998 | 1998 | 1999 | 1999 | 2000 | 2000 |
|  | Country | TUR | TUR | TUR | NLD | USA | TUR | Caucasian |
|  | Gender | F | F | M | M | F | F | F |
|  | Age of onset | 10m | 2m | 10m | 1m | 1m | 2.3y | 2y |
| Presenting symptom | Diarrhea | Yes | Yes | Yes | Yes | Yes | Yes | Yes |
|  | Vomiting | Yes | Yes | Yes | Yes | Yes | NM | NM |
|  | Nonimmune  Hydrops | NM | NM | NM | NM | Yes | Yes | NM |
|  | Hepatomegaly | Yes | Yes | NM | NM | Yes | NM | Yes |
|  | Splenomegaly | NM | NM | NM | NM | NM | NM | Yes |
|  | Portal hypertension | NM | NM | NM | NM | NM | Yes | Yes |
|  | Failure to thrive | No | No | No | NM | No | NM | No |
|  | Intellectual disability | No | No | No | NM | NM | NM | NM |
|  | Seizures | NM | NM | NM | NM | Yes | NM | NM |
|  | Protein-loosing enteropathy | NM | NM | NM | Yes | NM | Yes | NM |
| Laboratory findings | Hepatic fibrosis | NM | Yes | Yes | Yes | Yes | Yes | NM |
|  | Elevated serum transaminases | Yes | Yes | Yes | Yes | Yes | NM | NM |
|  | Hypoalbuminemia | Yes | Yes | Yes | Yes | Yes | NM | NM |
|  | Hyperinsulinism | NM | NM | NM | NM | Yes | Yes | NM |
|  | Hypoglycemia | NM | NM | NM | NM | Yes | Yes | NM |
|  | Prolonged coagulation | NM | NM | NM | NM | Yes | NM | NM |
|  | Base change 1 | NA | NA | NA | NA | c.1253G>A | c.152T>C | c.457G>A |
|  | Protein change 1 | NA | NA | NA | NA | R418H | M51T | R152Q |
|  | Base change 2 | NA | NA | NA | NA | c.1253G>A | c.152T>C | c.457G>A |
|  | Protein change 2 | NA | NA | NA | NA | R418H | M51T | R152Q |
|  | Other | NA | NA | NM | NM | Nipples were inverted | NM | NM |
| Mannose treatment |  | NM | NM | NM | No | Yes | Yes | Yes |
| Outcome |  | NM | NM | NM | Death | Better | NM | NM |

**Supplementary Table 1 (continued)**

|  | Patient number | 15^[7]^ | 16^[8]^ | 17^[9]^ | 18^[9]^ | 19^[10]^ | 20^[11]^ | 21^[12]^ |
| --- | --- | --- | --- | --- | --- | --- | --- | --- |
| General information | Years of publication | 2000 | 2001 | 2001 | 2001 | 2001 | 2003 | 2004 |
|  | Country | Asia | Asia | USA | USA | AUS | FR | NLD |
|  | Gender | NM | F | M | F | NM | M | F |
|  | Age of onset | NM | 6m | 2y | 2y | 5m | 3m | 2m |
| Presenting symptom | Diarrhea | NM | Yes | Yes | Yes | Yes | Yes | Yes |
|  | Vomiting | NM | Yes | Yes | Yes | NM | No | Yes |
|  | Nonimmune  Hydrops | NM | NM | Yes | No | NM | NM | NM |
|  | Hepatomegaly | NM | Yes | Yes | Yes | NM | Yes | Yes |
|  | Splenomegaly | NM | No | NM | No | Yes | No | NM |
|  | Portal hypertension | NM | No | NM | No | NM | NM | NM |
|  | Failure to thrive | NM | Yes | No | No | Yes | Yes | No |
|  | Intellectual disability | NM | NM | No | No | No | No | NM |
|  | Seizures | NM | NM | NM | NM | NM | No | No |
|  | Protein-loosing enteropathy | NM | Yes | Yes | Yes | Yes | No | Yes |
| Laboratory findings | Hepatic fibrosis | NM | Yes | Yes | No | Yes | No | Yes |
|  | Elevated serum transaminases | NM | Yes | No | No | Yes | No | Yes |
|  | Hypoalbuminemia | NM | Yes | Yes | Yes | Yes | No | NM |
|  | Hyperinsulinism | NM | Yes | NM | No | NM | NM | NM |
|  | Hypoglycemia | NM | Yes | NM | No | NM | Yes | Yes |
|  | Prolonged coagulation | NM | Yes | NM | No | Yes | Yes | Yes |
|  | Base change 1 | c.656G>A | c.391G>A | c.636G>A | c.636G>A | NA | c.386A>G | NM |
|  | Protein change 1 | R219Q | D131N | R219Q | R219Q | NA | Y129C | NM |
|  | Base change 2 | c.748G>A | c.391G>A | c.419T>C | c.419T>C | NA | c.455G>A | NM |
|  | Protein change 2 | G250S | D131N | I140T | I140T | NA | R152Q | NM |
|  | Other | NM | NM | Thrombosis | Thrombosis | NM | Thrombosis | NM |
| Mannose treatment |  | NM | Yes | NM | NM | NM | Yes | Yes |
| Outcome |  | NM | Better | Death | NM | NM | Better | Better |

**Supplementary Table 1 (continued)**

| Patient number | | 22^[12]^ | 23^[12]^ | 24^[13]^ | 25^[14]^ | 26^[15]^ | 27^[16]^ | 28^[17]^ |
| --- | --- | --- | --- | --- | --- | --- | --- | --- |
| General information | Years of publication | 2004 | 2004 | 2008 | 2008 | 2013 | 2014 | 2017 |
|  | Country | NLD | NLD | ES | NLD | NLD | SE | RUS |
|  | Gender | F | F | M | F | F | F | F |
|  | Age of onset | 2y | NM | 4m | 2y | 2y | NM | 9m |
| Presenting symptom | Diarrhea | No | No | Yes | Yes | Yes | No | Yes |
|  | Vomiting | No | No | NM | Yes | Yes | No | NM |
|  | Nonimmune  Hydrops | NM | NM | NM | NM | Yes | No | NM |
|  | Hepatomegaly | No | Yes | Yes | NM | Yes | No | NM |
|  | Splenomegaly | NM | NM | NM | NM | Yes | No | NM |
|  | Portal hypertension | NM | NM | NM | NM | Yes | No | NM |
|  | Failure to thrive | No | Yes | NM | NM | Yes | No | NM |
|  | Intellectual disability | No | No | No | NM | NM | No | NM |
|  | Seizures | No | No | No | NM | NM | No | NM |
|  | Protein-loosing enteropathy | No | No | Yes | NM | Yes | No | Yes |
| Laboratory findings | Hepatic fibrosis | No | Yes | NM | NM | Yes | No | Yes |
|  | Elevated serum transaminases | No | Yes | Yes | No | Yes | No | NM |
|  | Hypoalbuminemia | No | No | Yes | NM | Yes | No | Yes |
|  | Hyperinsulinism | NM | NM | NM | NM | NM | No | NM |
|  | Hypoglycemia | No | No | Yes | NM | NM | No | Yes |
|  | Prolonged coagulation | Yes | Yes | Yes | Yes | Yes | No | Yes |
|  | Base change 1 | NM | NM | c.656G>A | c.656G>A | c.455G>A | c.656G>A | c.655C>T |
|  | Protein change 1 | NM | NM | R219Q | R219Q | R152Q | R219Q | R219W |
|  | Base change 2 | NM | NM | 166-167  insC | c.656 G>A | c.41 A>C | c.656G>A | c.655C>T |
|  | Protein change 2 | NM | NM | R56fs | R219Q | Q14P | R219Q | R219W |
|  | Other | NM | NM | NM | Thrombosis | HE | No | Thrombosis |
| Mannose treatment |  | Yes | No | Yes | Yes | Yes | No | Yes |
| Outcome |  | Better | Death | Better | Better | LT | NA | Better |

**Supplementary Table 1 (continued)**

| Patient number | | 29^[17]^ | 30^[18]^ | 31^[19]^ | 32^[20]^ | 33^[21]^ | 34^[22]^ | 35^[23]^ |
| --- | --- | --- | --- | --- | --- | --- | --- | --- |
| General information | Years of publication | 2017 | 2017 | 2018 | 2018 | 2019 | 2019 | 2020 |
|  | Country | RUS | AE | TUR | ES | CN | CN | FR |
|  | Gender | M | F | F | F | M | F | M |
|  | Age of onset | 10m | 8m | 10m | 12y | 2y | 1y | 6m |
| Presenting symptom | Diarrhea | Yes | NM | Yes | Yes | Yes | Yes | Yes |
|  | Vomiting | NM | NM | Yes | NM | NM | NM | NM |
|  | Nonimmune  Hydrops | NM | No | NM | NM | NM | No | NM |
|  | Hepatomegaly | NM | Yes | Yes | NM | Yes | Yes | Yes |
|  | Splenomegaly | NM | NM | Yes | NM | Yes | No | No |
|  | Portal hypertension | NM | NM | NM | NM | Yes | NM | NM |
|  | Failure to thrive | Yes | No | No | NM | NM | No | No |
|  | Intellectual disability | NM | NM | No | NM | NM | No | No |
|  | Seizures | NM | NM | NM | NM | NM | NM | No |
|  | Protein-loosing enteropathy | Yes | NM | NM | NM | Yes | NM | No |
| Laboratory findings | Hepatic fibrosis | NM | NM | NM | NM | Yes | No | No |
|  | Elevated serum transaminases | Yes | Yes | Yes | NM | Yes | No | NM |
|  | Hypoalbuminemia | Yes | Yes | NM | Yes | Yes | NM | NM |
|  | Hyperinsulinism | NM | Yes | Yes | NM | NM | NM | NM |
|  | Hypoglycemia | NM | Yes | Yes | NM | NM | No | Yes |
|  | Prolonged coagulation | Yes | NM | Yes | Yes | Yes | No | NM |
|  | Base change 1 | c.1252C>T | NM | c.413T>C | c.1193T>C | c.1043T>C | c.391G>A | c.842-844del |
|  | Protein change 1 | R418C | A288V | M138T | I398T | I1348T | D131N | G281del |
|  | Base change 2 | c.1252C>T | NM | c.413T>C | c.656 G>A | c.1043T>C | c.455G>A | c.466G>A |
|  | Protein change 2 | R418C | A288V | M138T | A219G | I1348T | R152Q | E156K |
|  | Other | Yes | NM | NM | Thrombosis | NM | NM | NM |
| Mannose treatment |  | Yes | No | Yes | Yes | NM | Yes | Yes |
| Outcome |  | Better | Worse | Better | Better | Death | Better | Better |

**Supplementary Table 1 (continued)**

| Patient number | | 36^[23]^ | 37^[24]^ | 38^[23]^ | 39^[23]^ | 40^[23]^ | 41^[23]^ |
| --- | --- | --- | --- | --- | --- | --- | --- |
| General information | Years of publication | 2020 | 2020 | 2020 | 2020 | 2020 | 2020 |
|  | Country | FR | TUR | FR | FR | FR | FR |
|  | Gender | M | F | F | F | F | M |
|  | Age of onset | 3m | 5m | 3m | 20d | 3m | 2m |
| Presenting symptom | Diarrhea | Yes | Yes | Yes | Yes | Yes | Yes |
|  | Vomiting | NM | NM | Yes | Yes | NM | NM |
|  | Nonimmune  Hydrops | NM | No | NM | Yes | NM | NM |
|  | Hepatomegaly | Yes | Yes | Yes | Yes | Yes | Yes |
|  | Splenomegaly | Yes | NM | Yes | No | Yes | Yes |
|  | Portal hypertension | NM | NM | NM | NM | NM | NM |
|  | Failure to thrive | No | Yes | Yes | Yes | No | No |
|  | Intellectual disability | No | No | No | No | No | No |
|  | Seizures | No | NM | No | No | No | No |
|  | Protein-loosing enteropathy | Yes | Yes | Yes | Yes | Yes | Yes |
| Laboratory findings | Hepatic fibrosis | No | Yes | No | No | No | No |
|  | Elevated serum transaminases | NM | NM | Yes | Yes | NM | NM |
|  | Hypoalbuminemia | Yes | Yes | Yes | Yes | Yes | Yes |
|  | Hyperinsulinism | NM | Yes | NM | NM | NM | NM |
|  | Hypoglycemia | Yes | Yes | Yes | Yes | NM | No |
|  | Prolonged coagulation | NM | Yes | Yes | NM | NM | NM |
|  | Base change 1 | c.304C>T | c.1193T>C | c.764A>G | c.884G>A | c.655C>T | c.973delC |
|  | Protein change 1 | S102L | I398T | Y255C | R295H | R219W | L325S*24 |
|  | Base change 2 | c.413 T>C | c.1193T>C | c.1193T>C | c.884G>A | c.1252C>T | c.1193T>C |
|  | Protein change 2 | M138T | I398T | I398T | R295H | R418C | I398T |
|  | Other | Thrombosis | Abnormal fat distribution | Thrombosis | NM | NM | NM |
| Mannose treatment |  | No | NM | Yes | Yes | Yes | Yes |
| Outcome |  | Death | NM | Better | Better | Better | Better |

**Supplementary Table 1 (continued)**

| Patient number | | 42^[23]^ | 43^[23]^ | 44^[25]^ | 45^[26]^ | 46^[26]^ | 47^[27]^ |
| --- | --- | --- | --- | --- | --- | --- | --- |
| General information | Years of publication | 2020 | 2020 | 2020 | 2020 | 2020 | 2021 |
|  | Country | FR | FR | GER | EG | EG | FR |
|  | Gender | F | M | F | M | M | F |
|  | Age of onset | 6m | 10d | 15y | 2.5y | 1.4y | 4y |
| Presenting symptom | Diarrhea | Yes | Yes | No | Yes | Yes | NM |
|  | Vomiting | NM | NM | Yes | NM | NM | NM |
|  | Nonimmune  Hydrops | NM | NM | NM | Yes | No | NM |
|  | Hepatomegaly | Yes | Yes | No | Yes | Yes | Yes |
|  | Splenomegaly | Yes | Yes | No | Yes | Yes | NM |
|  | Portal hypertension | NM | NM | No | NM | Yes | NM |
|  | Failure to thrive | No | No | No | Yes | Yes | NM |
|  | Intellectual disability | No | No | NM | No | No | NM |
|  | Seizures | No | No | NM | NM | NM | NM |
|  | Protein-loosing enteropathy | Yes | Yes | No | Yes | Yes | NM |
| Laboratory findings | Hepatic fibrosis | No | No | No | NM | NM | NM |
|  | Elevated serum transaminases | NM | NM | No | Yes | Yes | No |
|  | Hypoalbuminemia | NM | NM | No | Yes | Yes | NM |
|  | Hyperinsulinism | NM | NM | NM | NM | NM | Yes |
|  | Hypoglycemia | Yes | Yes | No | No | Yes | Yes |
|  | Prolonged coagulation | NM | NM | NM | No | NM | NM |
|  | Base change 1 | c.1193T>C | c.884G>A | c.655C>T | c.487A>T | c.884G>A | NM |
|  | Protein change 1 | I398T | R295H | R219W | K163X | R295H | NM |
|  | Base change 2 | c.1193T>C | c.884G>A | c.1178G>C | c.656G>A | c.1193T>C | NM |
|  | Protein change 2 | I398T | R295H | G393A | R219Q | I398T | NM |
|  | Other | NM | PDA | ataxia | NM | Thrombosis,  Irritability | Thrombosis |
| Mannose treatment |  | Yes | Yes | NM | Yes | Yes | Yes |
| Outcome |  | Better | Better | NM | Better | Better | Better |

**Supplementary Table 1 (continued)**

| Patient number | | 48^[28]^ | 49^[28]^ | 50^[28]^ | 51^[29]^ | 52 |  |  |
| --- | --- | --- | --- | --- | --- | --- | --- | --- |
| General information | Years of publication | 2021 | 2021 | 2021 | 2021 | NA |  |  |
|  | Country | POL | POL | POL | USA | CN |  |  |
|  | Gender | NM | NM | NM | M | F |  |  |
|  | Age of onset | 2y | 12m | ＜2y | 2y | 4m |  |  |
| Presenting symptom | Diarrhea | NM | NM | NM | Yes | Yes |  |  |
|  | Vomiting | NM | NM | NM | NM | Yes |  |  |
|  | Nonimmune  Hydrops | NM | NM | NM | Yes | Yes |  |  |
|  | Hepatomegaly | Yes | Yes | NM | Yes | No |  |  |
|  | Splenomegaly | NM | NM | NM | NM | No |  |  |
|  | Portal hypertension | NM | NM | NM | NM | No |  |  |
|  | Failure to thrive | No | No | NM | NM | Yes |  |  |
|  | Intellectual disability | No | No | NM | NM | No |  |  |
|  | Seizures | NM | NM | NM | NM | No |  |  |
|  | Protein-loosing enteropathy | NM | NM | NM | Yes | Yes |  |  |
| Laboratory findings | Hepatic fibrosis | No | No | NM | NM | No |  |  |
|  | Elevated serum transaminases | Yes | Yes | NM | Yes | Yes |  |  |
|  | Hypoalbuminemia | NM | NM | NM | Yes | Yes |  |  |
|  | Hyperinsulinism | NM | NM | NM | Yes | Yes |  |  |
|  | Hypoglycemia | NM | NM | NM | Yes | No |  |  |
|  | Prolonged coagulation | NM | NM | NM | Yes | Yes |  |  |
|  | Base change 1 | c.1193T>C | c.656G>A | NM | c.656G>A | c.455G>T |  |  |
|  | Protein change 1 | I398T | A219G | NM | A219G | R152L |  |  |
|  | Base change 2 | c.1193T>C | c.656G>A | NM | c.602T>C | c.884G>A |  |  |
|  | Protein change 2 | I398T | A219G | NM | L201P | R295H |  |  |
|  | Other | NM | NM | NM | NM | No |  |  |
| Mannose treatment |  | Yes | Yes | Yes | Yes | No |  |  |
| Outcome |  | Better | Better | Better | Better | Better |  |  |

**Notes:** The country in the Table represents the country of origin of the patient. However, if the patient’s nationality was not referred to in the article, we defaulted to the country of the published article. **Abbreviations:** M, male; F, female; NM, not mentioned; y, year; w, week; m, month; HE, hepatic encephalopathy; LT, liver transplantation; PDA, patent ductus arteriosus

**Supplementary Table 2**. Significant mutation sites identified in the literature

| Mutation site | Type of mutations | Amino acid change | Number of reported | References |
| --- | --- | --- | --- | --- |
| c.656G>A | missense mutation | p.R219Q | 9 | [3], [7], [13], [14], [16], [20], [26], [28], [29] |
| c.884G>A | missense mutation | p.R295H | 8 | [2], [23], [26] |
| c.1193T>C | missense mutation | p.I398T | 7 | [20], [24], [23], [26], [28] |
| c.455G>A | missense mutation | p.R152Q | 3 | [11], [15], [22] |
| c.655C>T | missense mutation | p.R219W | 3 | [17], [23], [25] |
| c.636G>A | missense mutation | p.R219Q | 2 | [9] |
| c.419T>C | missense mutation | p.L140T | 2 | [9] |
| c.413T>C | missense mutation | p.M138T | 2 | [19], [23] |
| c.391G>A | missense mutation | p.D131N | 2 | [8], [22] |
| c.1252C>T | missense mutation | p.R418C | 2 | [23], [17] |
| c.1253G>A | missense mutation | p.R418H | 1 | [6] |
| c.152T>C | missense mutation | p.M51T | 1 | [7] |
| c.457G>A | missense mutation | p.R152Q | 1 | [7] |
| c.386A>G | missense mutation | p.Y129C | 1 | [11] |
| c.748G>A | missense mutation | p.G250S | 1 | [7] |
| c.487A>T | missense mutation | p.K163X | 1 | [26] |
| c.863C＞T | missense mutation | p.A288V | 1 | [18] |
| c.602T>C | missense mutation | p.L201P | 1 | [29] |
| c.764A>G | missense mutation | p.Y255C | 1 | [23] |
| c.304C>T | missense mutation | p.S102L | 1 | [23] |
| c.466G>A | missense mutation | p.E156K | 1 | [23] |
| c.1178G>C | missense mutation | p.G393A | 1 | [25] |
| c.455G>T | missense mutation | p.R152L | 1 | the current case |
| c.1043T>C | missense mutation | p.L348T | 1 | [21] |
| c.41A>C | missense mutation | p.Q14P | 1 | [15] |
| c.973delC | frameshift mutation | p.L325Sfs*24 | 1 | [23] |
| c.842_844del | deletion mutation | p.G281del | 1 | [23] |
| 166-167insC | frameshift mutation | R56fs | 1 | [13] |

**References**

[1] P.S. Pedersen, and I. Tygstrup, Congenital hepatic fibrosis combined with protein-losing enteropathy and recurrent thrombosis. Acta paediatrica Scandinavica 69 (1980) 571-574.

[2] S. Vuillaumier-Barrot, C. Le Bizec, P. de Lonlay, A. Barnier, G. Mitchell, V. Pelletier, C. Prevost, J.M. Saudubray, G. Durand, and N. Seta, Protein losing enteropathy-hepatic fibrosis syndrome in Saguenay-Lac St-Jean, Quebec is a congenital disorder of glycosylation type Ib. Journal of medical genetics 39 (2002) 849-851.

[3] R. Niehues, M. Hasilik, G. Alton, C. Körner, M. Schiebe-Sukumar, H.G. Koch, K.P. Zimmer, R. Wu, E. Harms, K. Reiter, K. von Figura, H.H. Freeze, H.K. Harms, and T. Marquardt, Carbohydrate-deficient glycoprotein syndrome type Ib. Phosphomannose isomerase deficiency and mannose therapy. The Journal of clinical investigation 101 (1998) 1414-1420.

[4] T.J. de Koning, L. Dorland, O.P. van Diggelen, A.M. Boonman, G.J. de Jong, W.L. van Noort, J. De Schryver, M. Duran, I.E. van den Berg, G.J. Gerwig, R. Berger, and B.T. Poll-The, A novel disorder of N-glycosylation due to phosphomannose isomerase deficiency. Biochemical and biophysical research communications 245 (1998) 38-42.

[5] D.M. Neele, C.M. Kneepkens, N.M. Verhoeven, and C. Jakobs, Retrospective diagnosis of carbohydrate-deficient glycoprotein syndrome type Ib. Journal of inherited metabolic disease 22 (1999) 936-937.

[6] D. Babovic-Vuksanovic, M.C. Patterson, W.F. Schwenk, J.F. O'Brien, J. Vockley, H.H. Freeze, D.P. Mehta, and V.V. Michels, Severe hypoglycemia as a presenting symptom of carbohydrate-deficient glycoprotein syndrome. The Journal of pediatrics 135 (1999) 775-781.

[7] E. Schollen, L. Dorland, T.J. de Koning, O.P. Van Diggelen, J.G. Huijmans, T. Marquardt, D. Babovic-Vuksanovic, M. Patterson, F. Imtiaz, B. Winchester, M. Adamowicz, E. Pronicka, H. Freeze, and G. Matthijs, Genomic organization of the human phosphomannose isomerase (MPI) gene and mutation analysis in patients with congenital disorders of glycosylation type Ib (CDG-Ib). Human mutation 16 (2000) 247-252.

[8] C.J. Hendriksz, P. McClean, M.J. Henderson, D.G. Keir, V.C. Worthington, F. Imtiaz, E. Schollen, G. Matthijs, and B.G. Winchester, Successful treatment of carbohydrate deficient glycoprotein syndrome type 1b with oral mannose. Archives of disease in childhood 85 (2001) 339-340.

[9] V. Westphal, S. Kjaergaard, J.A. Davis, S.M. Peterson, F. Skovby, and H.H. Freeze, Genetic and metabolic analysis of the first adult with congenital disorder of glycosylation type Ib: long-term outcome and effects of mannose supplementation. Molecular genetics and metabolism 73 (2001) 77-85.

[10] D.F. Kelly, A. Boneh, S. Pitsch, H. Gold, M. Fietz, P. Nelson, and M.R. Oliver, Carbohydrate-deficient glycoprotein syndrome 1b: a new answer to an old diagnostic dilemma. Journal of paediatrics and child health 37 (2001) 510-512.

[11] D. Penel-Capelle, D. Dobbelaere, J. Jaeken, A. Klein, M. Cartigny, and J. Weill, Congenital disorder of glycosylation Ib (CDG-Ib) without gastrointestinal symptoms. Journal of inherited metabolic disease 26 (2003) 83-85.

[12] G. Damen, H. de Klerk, J. Huijmans, J. den Hollander, and M. Sinaasappel, Gastrointestinal and other clinical manifestations in 17 children with congenital disorders of glycosylation type Ia, Ib, and Ic. Journal of pediatric gastroenterology and nutrition 38 (2004) 282-287.

[13] E. Martín Hernández, A.I. Vega Pajares, B. Pérez González, M.J. Ecay Crespo, F. Leal Pérez, J. Manzanares López-Manzanares, M. Ugarte Pérez, and C. Pérez-Cerdá Silvestre, [Congenital disorder of glycosylation type 1b. Experience with mannose treatment]. Anales de pediatria (Barcelona, Spain : 2003) 69 (2008) 358-365.

[14] R.Y. Tamminga, D.J. Lefeber, W.A. Kamps, and F.J. van Spronsen, Recurrent thrombo-embolism in a child with a congenital disorder of glycosylation (CDG) type Ib and treatment with mannose. Pediatric hematology and oncology 25 (2008) 762-768.

[15] M.C. Janssen, R.H. de Kleine, A.P. van den Berg, Y. Heijdra, M. van Scherpenzeel, D.J. Lefeber, and E. Morava, Successful liver transplantation and long-term follow-up in a patient with MPI-CDG. Pediatrics 134 (2014) e279-83.

[16] A. Helander, J. Jaeken, G. Matthijs, and G. Eggertsen, Asymptomatic phosphomannose isomerase deficiency (MPI-CDG) initially mistaken for excessive alcohol consumption. Clinica chimica acta; international journal of clinical chemistry 431 (2014) 15-18.

[17] A.V. Migali, K.A. Kazakova, N.V. Zhurkova, M. Mitish, and Y. Akoev, P14 Congenital disorder of glycosylation ib type (CDG IB): 2 cases of diagnostics and treatment, 8th Europaediatrics Congress jointly held with, The 13th National Congress of Romanian Pediatrics Society, 7–10 June 2017, Palace of Parliament, Romania, Paediatrics building bridges across Europe, 2017.

[18] A. Deeb, and A. Al Amoodi, A novel homozygous mutation in the mannose phosphate isomerase gene causing congenital disorder of glycation and hyperinsulinemic hypoglycemia in an infant. Clinical case reports 6 (2018) 479-483.

[19] P. Haznedar, and F.T. Eminoğlu, An overlooked case of a treatable hyperinsulinemic hypoglycemia: congenital glycosylation defect Type Ib. Turk pediatri arsivi 55 (2020) 79-81.

[20] M.E. de la Morena-Barrio, E. Wypasek, D. Owczarek, A. Miñano, V. Vicente, J. Corral, and A. Undas, MPI-CDG with transient hypoglycosylation and antithrombin deficiency. Haematologica 104 (2019) e79-e82.

[21] Liu J, Hao N, Zhou H, Tian T, Li C, Wang R, A case of congenital glycosylation disorder type Ib . Chinese Journal of Difficult and Complicated Cases 18 (2019) 2.

[22] Zeng J , Jiang L , Wang J , Deng C et al. Clinical and genetic analysis for congenital disturbance of glycosylation with MPI gene mutation[J]. Journal of Clinical Pediatrics 37 (2019) 3.

[23] M. Girard, C. Douillard, D. Debray, F. Lacaille, M. Schiff, S. Vuillaumier-Barrot, T. Dupré, M. Fabre, L. Damaj, A. Kuster, S. Torre, K. Mention, V. McLin, D. Dobbelaere, D. Borgel, E. Bauchard, N. Seta, A. Bruneel, and P. De Lonlay, Long term outcome of MPI-CDG patients on D-mannose therapy. Journal of inherited metabolic disease 43 (2020) 1360-1369.

[24] T.R. Zdemir, Congenital Disorder of Glycosylation: Clinical and Molecular Characteristics of 9 Patients from Turkey. Journal of Dr Behcet Uz Children s Hospital (2020).

[25] C. Mühlhausen, L. Henneke, L. Schlotawa, D. Behme, M. Grüneberg, J. Gärtner, and T. Marquardt, Mannose phosphate isomerase deficiency-congenital disorder of glycosylation (MPI-CDG) with cerebral venous sinus thrombosis as first and only presenting symptom: A rare but treatable cause of thrombophilia. JIMD reports 55 (2020) 38-43.

[26] T.Y. Abdel Ghaffar, B.G. Ng, S.M. Elsayed, S. El Naghi, S. Helmy, N. Mohammed, A. El Hennawy, and H.H. Freeze, MPI-CDG from a hepatic perspective: Report of two Egyptian cases and review of literature. JIMD reports 56 (2020) 20-26.

[27] E. Lebredonchel, S. Duvet, C. Douillard, F. Foulquier, and A. Klein, Variation of the serum N-glycosylation during the pregnancy of a MPI-CDG patient. JIMD reports 62 (2021) 22-29.

[28] A. Bogdańska, P. Lipiński, P. Szymańska-Rożek, A. Jezela-Stanek, D. Rokicki, P. Socha, and A. Tylki-Szymańska, Clinical, biochemical and molecular phenotype of congenital disorders of glycosylation: long-term follow-up. Orphanet journal of rare diseases 16 (2021) 17.

[29] R. Mullin, H. Baines, and K. Connelly, Congenital Disorder of Glycosylation 1b: Rare cause of hyperinsulinemic hypoglycemia treated with oral mannose therapy. Hormone Research in Paediatrics 94 (2021) 62-63.
